# AbMiner: A bioinformatic resource on available monoclonal antibodies and corresponding gene identifiers for genomic, proteomic, and immunologic studies

**DOI:** 10.1186/1471-2105-7-192

**Published:** 2006-04-06

**Authors:** Sylvia M Major, Satoshi Nishizuka, Daisaku Morita, Rick Rowland, Margot Sunshine, Uma Shankavaram, Frank Washburn, Daniel Asin, Hosein Kouros-Mehr, David Kane, John N Weinstein

**Affiliations:** 1 Genomics and Bioinformatics Group, Laboratory of Molecular Pharmacology, National Cancer Institute, National Institutes of Health, Bethesda, USA; 2 Center for Information Technology, National Institutes of Health, Bethesda, USA; 3 SRA International, 4300 Fair Lakes Court, Fairfax, USA; 4 University of California at Los Angeles, Department of Ecology and Evolutionary Biology, Los Angeles, USA; 5 Molecular Translational Technology, Molecular Therapeutics Program, Center for Cancer Research, National Cancer Institute, National Institutes of Health, Bethesda, USA; 6 Laboratory of Proteomics and Analytical Technologies, Research Technology Program, National Cancer Institute, SAIC-Frederick, Frederic, USA; 7 Department of Pathology II, National Defense Medical College, Namiki 3-2, Tokorozawa, Japan; 8 Harvard University, Cambridge, USA; 9 Washington University, St. Louis, USA; 10 Department of Anatomy, University of California, San Francisco, San Francisco,

## Abstract

**Background:**

Monoclonal antibodies are used extensively throughout the biomedical sciences for detection of antigens, either i*n vitro *or *in vivo*. We, for example, have used them for quantitation of proteins on "reverse-phase" protein lysate arrays. For those studies, we quality-controlled > 600 available monoclonal antibodies and also needed to develop precise information on the genes that encode their antigens. Translation among the various protein and gene identifier types proved non-trivial because of one-to-many and many-to-one relationships. To organize the antibody, protein, and gene information, we initially developed a relational database in Filemaker for our own use. When it became apparent that the information would be useful to many other researchers faced with the need to choose or characterize antibodies, we developed it further as AbMiner, a fully relational web-based database under MySQL, programmed in Java.

**Description:**

AbMiner is a user-friendly, web-based relational database of information on > 600 commercially available antibodies that we validated by Western blot for protein microarray studies. It includes many types of information on the antibody, the immunogen, the vendor, the antigen, and the antigen's gene. Multiple gene and protein identifier types provide links to corresponding entries in a variety of other public databases, including resources for phosphorylation-specific antibodies. AbMiner also includes our quality-control data against a pool of 60 diverse cancer cell types (the NCI-60) and also protein expression levels for the NCI-60 cells measured using our high-density "reverse-phase" protein lysate microarrays for a selection of the listed antibodies. Some other available database resources give information on antibody specificity for one or a couple of cell types. In contrast, the data in AbMiner indicate specificity with respect to the antigens in a pool of 60 diverse cell types from nine different tissues of origin.

**Conclusion:**

AbMiner is a relational database that provides extensive information from our own laboratory and other sources on more than 600 available antibodies and the genes that encode the antibodies' antigens. The data will be made freely available at

## Background

Antibodies are used as tools throughout biomedical science, and they are, increasingly, being incorporated into clinical practice in such specialties as rheumatology, oncology, and infectious diseases [1]. They are also finding more and more application in the new high-throughput biotechnologies such as antibody and protein lysate microarrays [2-8]. As a consequence of that increased prominence and range of application, antibody reagents (particularly monoclonals) are being made available to the researcher commercially in increasing numbers. However, some of them do not have the right affinity, specificity, or other characteristics for a particular application, creating a problem and, often, wasted effort for end-users [8].

That was the case when our laboratory began the project that motivated us to develop AbMiner: 'reverse-phase' protein lysate microarray profiling of the 60 human cancer cell lines (the NCI-60) used since 1990 by the U.S. National Cancer Institute's Developmental Therapeutics Program to screen > 100,000 chemical compounds (plus natural products) for anticancer activity [9,10]. In 2001, Paweletz, et al. [11] introduced 'reverse phase' protein lysate microarrays (henceforth, called 'lysate arrays' here), in combination with laser capture microdissection and robotic spotting technology. For the NCI-60 project, we [12] then developed higher density lysate arrays that incorporated all 60 cell lines plus controls, each at 10 serial two-fold dilutions to achieve wide dynamic range and good reproducibility (17% coefficient of variation) in profiling of protein levels across the cell types. Antibodies were used to quantify protein on the arrays using a Catalyzed Signal Amplification method (DAKO Cytomation, Carpenteria, CA, USA). We obtained more than 600 commercially available monoclonal antibodies to find ones suitable for the purpose. Before application to the arrays, we screened the antibodies by Western blot against a pool of the NCI-60 lysates (equal amounts from each cell type). Since the pool included cancer cell lines from 9 different tissues of origin, it served as an extensive (though not exhaustive) sampling of human protein antigens.

To record and monitor the validation process, we programmed a relational database that included the results as well as correlative meta-data on each antibody reagent. It became apparent, however, that the database (which we later called AbMiner) would be valuable to a much broader community of antibody users. We therefore decided to develop it further as a public resource. For the 635 antibodies included to date, the user can browse the information or search by antibody name or by any of 18 other features (Table 1). Although each investigator will presumably want to vet antibodies for his or her particular application, quality-control data and other information in AbMiner can minimize time spent searching for usable antibody reagents.

In addition, AbMiner provides a connection to other 'omic' data [13] by matching each antibody with the target antigen's corresponding DNA and RNA identifiers. We were initially motivated to translate antibody names to gene symbols because of studies in which we were correlating protein and mRNA expression from microarrays for biomarker discovery [14]. The gene information in AbMiner was later expanded to include a variety of genomic and proteomic identifiers. Using our MatchMiner program package [15], a tool for batch-translation of identifiers, we matched each antibody to its antigen's gene symbol. The corresponding gene name, UniGene cluster ID, LocusLink ID, and RefSeq were then identified using MatchMiner and other tools [15-22], with careful manual curation. AbMiner provides link-outs from gene identifiers to the corresponding entries in various public resources (LocusLink, GeneCards, etc.), as well as to other antibody databases. By providing a means to search by DNA, RNA, or protein, AbMiner facilitates the integration of genomic, transcriptomic, and proteomic information.

## Construction and content

### Antibody validation

Monoclonal antibodies directed against protein antigens were obtained from many different commercial sources (listed in the AbMiner program itself), with no particular selection criteria except that antibodies directed against very small (~ 10 kDa) or very large (> 350 kDa) proteins were excluded. Species recognized included human, mouse, rat, dog, chicken, frog, and others. Each vial of antibody was assigned a unique AbMiner identification code number so that screening of the particular vial could be tracked. Pertinent information indicated in the schema in Table 1 was also recorded.

**Figure 1:**
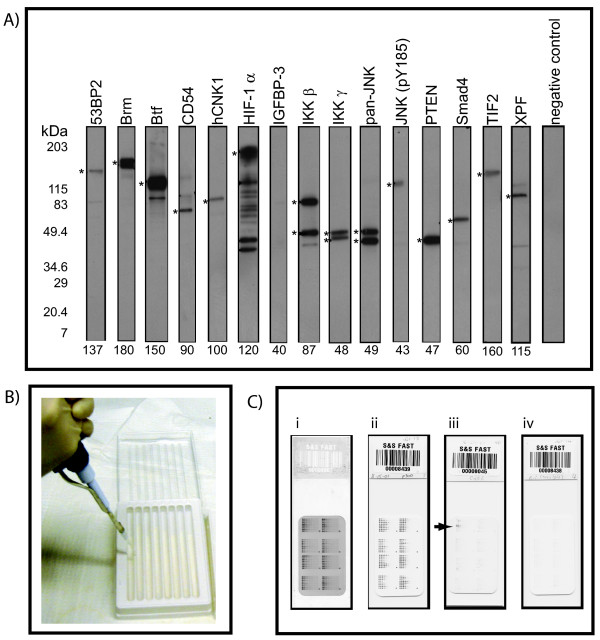
Antibody screening and NCI-60 'reverse-phase' protein lysate microarrays. A) Sixteen 4-mm-wide nitrocellulose strips from a nitrocellulose membrane blot, each used to test a different antibody. Each blot was classified by the criteria described in the text. Asterisks indicate predominant band(s). Eight blots (53BP2, Brm, Btf, hCNK1, PTEN, Smad4, TIF2, and XPF) were classified as *single band and the expected molecular weight*. Four blots (HIF-1 α, IKK β, IKK γ, and pan-JNK) were classified as *multiple bands*. Two blots (CD54, and JNK pY185) were classified as *wrong molecular weight*. One blot (IGFBP-3) was classified as *no band*. All band results from this Western blotting were entered into AbMiner. It should be noted that the results in this figure may not correspond to the band results in AbMiner because the latter are sometimes updated after additional testing. B) Miniature incubation chambers for 4-mm strips used for incubations with primary and then secondary antibodies. C) Reverse-phase lysate arrays. Each row consists of 10 two-fold dilutions of an NCI-60 cell line or a control pool consisting of all 60 cell lines. Concentrated pool was spotted at the bottom-right corner of each field to serve as a registration mark for image processing. i) lysate array stained for total protein with SYPRO Ruby. ii) lysate array incubated with an antibody for p300, a protein ubiquitously expressed in the NCI-60. iii) CDK2 expression across 60 cell lines. Only one cell line shows visible expression (arrow). Nonetheless, expression of CDK2 was still detected as a single band by Western blot iv) Negative control. The primary antibody was replaced by anti-*Aspergillus niger *glucose oxidase IgG_1_, which does not recognize any human antigen.

To validate antigen-specificity by Western blot against a wide range of human antigens, we harvested cell lysates from each of the NCI-60 cell lines as described previously [23,24] and generated a standard pool (NCI-60 pool) containing approximately equal amounts of each of the 60 lines. Included in the pool were leukemias of several lineages, melanomas, and cancers of breast, ovary, prostate, colon, lung, kidney, and central nervous system. After electrophoresis and blotting, each gel yielded 16 four-mm wide nitrocellulose strips, each of which was used to screen a different antibody (Fig. 1a). To increase the likelihood of detecting low-abundance proteins, we loaded the gels quite heavily so that each small strip contained 0.6–1.3 μg of pool protein. Up to 64 antibodies were screened simultaneously by incubating each strip in its own miniature incubation chamber (BioRad, Hercules, CA, USA) (Fig. 1b).

The Western blot results were classified as follows: (a) *single band*: one predominant band, at the expected molecular weight; (b) *multiple bands*: extra bands remaining with a 5-second or longer film exposure; (c) *wrong molecular weight*: predominant band or bands at unexpected molecular weight(s); and (d) *no band*. Antibodies were Western-blotted up to three or four times if required to obtain clear results.

We focused on Western blot analysis and designed the screening process as we did because of the specific requirements for application of antibodies on reverse-phase lysate arrays. Antibodies that recognize an epitope from more than one protein (or isoform) can be used for detection and quantitation of proteins on a Western blot as long as any extraneous bands have different effective molecular weights and would show up as separate bands. The lysate array, in contrast, is effectively a multiplexed dot blot; the signal from each spot on the array is the summation of specific and non-specific binding of the antibody. Therefore, for the lysate arrays we used only screened antibodies that produced a single predominant band by Western at the expected molecular weight. However, antibodies that produced multiple bands may still be useful for other applications, so information on them is retained in AbMiner. Other types of quality control data (for example, based on immunoprecipitation, immunohistochemistry, or flow cytometry) may be most pertinent to other types of applications. AbMiner is extensible in that data fields can be added to accommodate and present such information.

**Figure 2:**
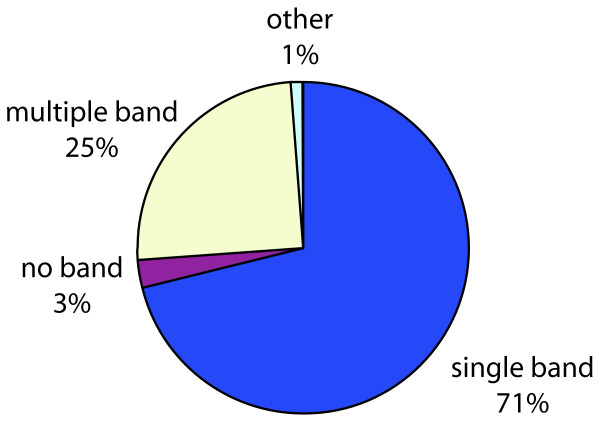
Summary of Western blot results for the first 441 antibodies screened (and currently in AbMiner). The majority of antibodies (71%) produced a single predominant band.

As indicated in Fig. 2, approximately 70% of the commercially available antibodies we screened produced a single predominant band against the NCI-60 lysate pool; only 4% resulted in absent or questionable bands. Some antibodies that produced a single band by Western blot were expressed in only one or two cell lines out of the 60 on the NCI-60 lysate arrays (Fig. 1c), indicating that the validation method is quite sensitive. Since each cell line provides about 1/60^th ^of the pool's protein, we cannot rule out the possibility that a relatively faint extraneous band on the western would reflect a grossly incorrect target protein value for one or two cell types on the array. To check that possibility would require doing 60 times as many Westerns (or pools of small numbers of cell types perhaps). In principle, that would be desirable, but the uncertainties of the present quality control procedure are still considerably less than those inherent in many other transcriptomic and proteomic profiling technologies. As noted previously, the user will presumably reassess candidate antibodies on his or her particular experimental system in any case.

### Gene information

AbMiner's Gene Information database provides translation among different data platforms and makes it possible for the user to search by proteomic, transcriptomic, or genomic identifiers. To find the intersection between data sets from different platforms – such as cDNA [25,26] and oligonucleotide microarrays [27]– one generally must translate from one type of unique identifier to another [15]. Finding an antibody that corresponds to a particular gene can be problematic because many commercially available antibodies do not have unique, universally used names that represent the target gene product.

**Figure 3:**
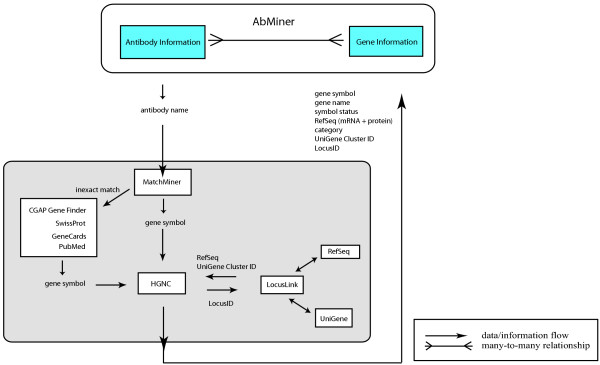
Pipeline for matching antibodies to gene identifiers. Antibodies were matched to corresponding gene symbols using MatchMiner or a combination of other resources if the symbol was not immediately retrievable with MatchMiner. The translation from symbol to LocusID was obtained from the Human Genome Nomenclature Committee (HGNC). LocusLink ftp -downloadable files provided UniGene Cluster Ids and RefSeqs. We then used the LocusID to join to the HGNC file, after which all identifiers could be mapped back to the antibody originally input into MatchMiner. The often-complex, many-to-many relationships between genes and antibodies are described in the text.

To match antibodies names with the appropriate identifiers of the corresponding gene, we used MatchMiner [15]. MatchMiner translates among various gene and protein identifier types, including HUGO name, common names, aliases, chromosome locations, GenBank accession numbers, Affymetrix identifiers, and IMAGE clone IDs (Fig. 3). For antibodies whose names could not immediately be translated with MatchMiner, other resources were used to cross-check and select the appropriate symbol [16-22,28,29]. The gene symbols were then matched with the other identifiers as described above. The final MatchMiner output included the gene name and symbol, the HUGO approval status of the symbol, mRNA and protein RefSeqs, UniGene Cluster IDs, and LocusIDs. Through a many-to-many relationship with the Antibody Information File in AbMiner, any antibody can be associated with its appropriate gene identifiers and *vice versa*. That feature of AbMiner (see Table 1) facilitates navigation among antibody, antigen, protein, DNA, and RNA data, allowing for integration of information from disparate data sets.

### System design and implementation

AbMiner is a relational database comprised of two major components: (i) a data entry module constructed using FileMaker Pro5.0™ (Santa Clara, CA USA) and used by our team for data entry as well as for detailed tracking of the antibody validation process.

(ii) a web application for sharing the various types of information on antibodies, antigens, and genes with the research community. The web application, written principally in Java, leverages a variety of available resources: MySQL as the database engine; Hibernate to map the objects into the database; JSP, Struts, and Tiles to render the user interface; and JUnit and HTTPUnit for testing individual programming units and the overall system. In addition to providing the web user interface, we have defined a simple HTTP specification that facilitates linkage of other applications into AbMiner. The web application was constructed under the "Agile Development" paradigm, which encourages close, iterative interaction between user/tester/motivators of the package (biologists) and software engineers [30]. That interaction, and the continuing revision of specs that the agile process encourages, ensure that AbMiner will serve broad needs of biological researchers. It has been received enthusiastically in extensive beta-testing.

Both components of AbMiner have the same underlying model, which includes three main modules: Antibody, Screening, and Gene. AbMiner uses a relational database approach to manage the complex relationships among those elements. The relationships are generally not one-to-one. For example, a given gene often codes for different splice variants, which may or may not be recognized by the same antibody. Conversely, multiple antibodies from different vendors or hybridoma clones may target a protein encoded by a single gene. An additional complication is that, because of the continuing re-annotation of the human genome, some identifiers are not unique or constant. That dynamic process is exemplified by retired or relocated UniGene clusters that can sometimes result in more than one UniGene Cluster ID or LocusID entry for the same gene. By constructing AbMiner as a relational database, we have been able to organize and update those one-to-many, many-to-one, and many-to-many relationships.

Currently, AbMiner is populated with screening data generated by our own laboratory, but we plan to incorporate data from other studies and repositories when available. We also plan to put data entry pages on the web component for input of screening information from other investigators in the research community who wish to contribute (with appropriate attribution)

### AbMiner link-outs

The identifiers described in the last section perform an additional function by serving as link-outs to their respective entries in LocusLink, UniGene, GeneCards, Entrez's RefSeq, and our MedMiner program. AbMiner also provides links to the Mammalian Phosphorylation Resource (MPR) [31], a web site that contains sequence information for phosphorylation sites recognized with specificity by commercial antibodies, and links to the Clinical Proteomics Databank [32], which provides a list of phospho-specific antibodies tested and used in the Clinical Proteomics Program of the NCI. Other public and commercial antibody databases, such as the Antibody Resource page [33]and Abcam [34], are also linked. Finally, AbMiner will serve as the central public database for an antibody repository planned by the NCI Center for Cancer Research (CCR).

## Utility and discussion

### AbMiner applied to molecular biomarker identification

As already noted, development of AbMiner was motivated by our need to organize information on antibodies for lysate array studies, and it has proved itself an almost indispensable tool in that respect. Particularly important is the information on the correspondence between antibody names, antigen names, and the variety of gene identifier types. We were able, for example, to address the question of similarities and differences between mRNA and protein expression profiles across the NCI-60 [12]. Identifiers of proteins quantitated on lysate arrays were matched with identifiers of transcripts assessed on spotted cDNA arrays (i.e., Image Clone Ids) and Affymetrix oligonucleotide arrays (i.e., Affymetrix Ids) using MatchMiner [15] and AbMiner. A central, unexpected finding was that cell-structure-related proteins showed higher correlation between protein and mRNA levels across the 60 cell lines than did non-cell-structure-related proteins [12]. Using the annotations and translation capabilities in AbMiner, those analyses have now been extended to 89 proteins detected on the lysate arrays by 154 different antibodies (Shankavaram et al. manuscript in preparation).

We have also applied the resources of AbMiner to the identification of molecular biomarkers at the protein level. For one such study, we developed a multi-step "integromic" protocol [14,30], which included: (a) identification of candidate markers using cDNA microarrays; (b) re-sequencing of candidate clones; (c) corroboration of the candidates' expression patterns from the cDNA microarray using Affymetrix oligonucleotide chips; (d) protein expression analysis using reverse-phase protein lysate arrays; and (e) prospective validation of candidate biomarkers on tissue microarrays consisting of hundreds of tumor samples. With that algorithm we identified villin and moesin as molecular markers that distinguish between colon and ovarian adenocarcinomas. Those cancer types can be difficult to distinguish in a few percent of metastatic or disseminated lesions in the abdomen, and the differential diagnosis is important because it determines what drugs will be used for therapy. Our protocol was successful in that case, but it depended on the availability and effective screening of quality antibodies for identification of diagnostic markers at the protein level on the lysate and tissue arrays. AbMiner gene identifiers will help other investigators in similar searches for molecular markers at the protein level, even when the search has begun with genomic databases. Because AbMiner provides extensive information for over 600 validated antibodies, the transcriptional signature of a gene can often be corroborated directly at the protein level.

The protein-mRNA expression studies are continuing, with promise. More than 500 of the 635 antibodies currently in AbMiner have UniGene Cluster IDs that match those from four different NCI-60 microarray platforms (see Table 2 and 3), providing a foundation for cross-comparison, for validation studies, and for hypothesis generation. AbMiner is contributing both to the selection of antibodies and to the crucial link between protein and transcriptional data.

### Comparison with other antibody databases

AbMiner is certainly not the most comprehensive in terms of numbers of antigens or antibodies covered. In that regard, the classic source is Linscott's Directory [35]. The Advanced Type Culture Collection (ATCC) [36] keeps listings of large numbers of their available cell lines, including hybridomas, and the Antibody Resource Page [33] and Abcam [34] provide useful databases on antibodies. AbMiner includes links to all of those sources. A number of companies provide databases of the antibodies they sell, but those will not be reviewed here. AfCS Signaling Gateway provides [38] provides information on 138 proteins (principally in the signaling pathways) and antibodies against them. Western Blot quality control information, generally on one or a few cell lines, is included. Exactantigen [39] provides gene-specific and species-specific information on antibodies, with links to manufacturers' data sheets. The useful Human Protein Atlas [40,41] features immunohistochemical images for a variety of newly generated and other antibodies, complementing the focus of AbMiner. There are also a number of specialized antibody collections (e.g., on 3-D structures of antibodies or on neurological or HIV-related reagents) [42,43], but none that we have seen present ranges of information similar to that of AbMiner. It would be well beyond the scope of this article to review those databases, but a number of them are described, with outlinks, by Linscott's. AbMiner's database will continue to expand, but not with the intention of competing with Linscott's in coverage.

Overall, to the best of our knowledge, none of the other sources have the range of information types on the antibodies, the antigens, the vendors, and the antigen's genes that AbMiner does, and none of them give the type of multiple-tissue Western blot specificity data or protein microarray data that are compiled in AbMiner. Insofar as we have found, any Western blot results given in the other databases had been obtained against single cell types. The quality control criteria represented in AbMiner are stricter and more comprehensive in that we have validated the antibodies by Western blot against a pool representing a wide range of cancer cell lines from nine different organs of origin and from different cell lineages. Non-specificities showed up in that more rigorous testing when they didn't in testing against individual cell types.

AbMiner has unique relational characteristics for dealing with the one-to-many, many-to-one, and many-to-many relationships among antibody reagents, their antigens, and the genes of those antigens. Through the use of MatchMiner [15], supplemented by manual curation from additional bioinformatic resources, AbMiner gives a useful range of gene identifier types not otherwise easy for the casual user to find. The Antibody Resource Page provides a listing of "databases/software" on immunological reagents [33], but none of those listed have major overlap with AbMiner in terms of their program and search capabilities. We are currently using the structure of AbMiner as template for an analogous database on siRNA reagents.

We developed AbMiner as we did to provide the type of information needed for "integromic" [30,44] studies of the type described above for biomarker identification – that is, for the integration of different types of molecular data at the DNA, RNA, protein, and functional levels. But the program is also being found useful (in beta-testing) by researchers with simpler aims: e.g., those who simply want to find the right antibody for an assay.

## Conclusion

**Figure 4:**
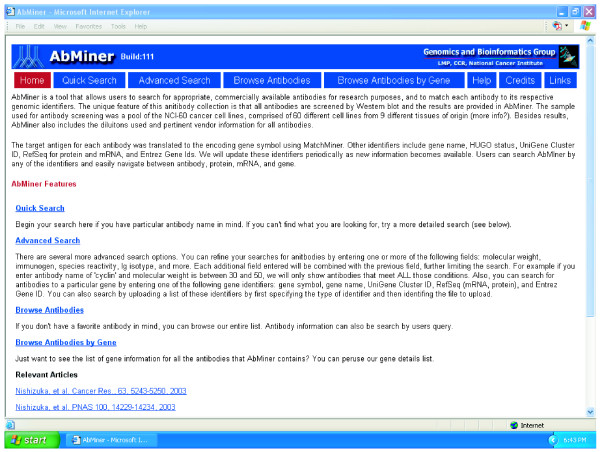
Screen shot of the AbMiner home page, at . All search and browse links are shown, along with brief descriptions of each function. *[Note to the Editor and Reviewers: The screen shot will be updated to reflect its content at the time of publication.]*

AbMiner (Fig. 4) is a broadly useful, user friendly resource for finding validated antibodies or for connecting antibodies with gene identifiers and other types of genomic/proteomic information. The user can browse or search by multiple, full Boolean criteria for an antibody of interest or look up corresponding antibodies from a list of genes denoted by any of a variety of different identifiers. The database is currently populated with > 600 available antibodies plus information on their quality control and specificity against a pool of antigens from 60 diverse cell lines in the NCI-60 panel. Also included are quantitative protein profiles across the NCI-60 cell lines for some of the antibodies, as well as extensive antibody, protein, and gene identifier translations and link-outs to a variety of public bioinformatic resources. AbMiner will enhance the ability of the research community to find suitable antibodies and to link proteomics, transcriptomics, and genomics in 'integromic' [30,44] studies.

## Availability and requirements

AbMiner is freely accessible to both public and private sector users at . Also available there for batch downloading are the quality control results and lysate array data for screened antibodies. They will be updated as new antibodies are tested [28]. Also available there is a detailed protocol for the Western blot screening. Gene Information FILES will be updated regularly. As a Java implementation, AbMiner is browser-, operating system-, and platform-independent.

## Authors' contributions

S.M. Major spearheaded the antibody quality control studies and software development and wrote the manuscript. S. Nishizuka designed the project and led the team in the lysate array experiments. D. Morita, F. Washburn, D. Asin, and H. Kouros-Mehr provided extensive experimental results. R. Rowland coded a prototype of the package in File Maker Pro. M. Sunshine and D. Kane developed the MySQL web interface and refactored the program package as a Java implementation. U. Shankavaram analyzed and organized the protein array data. J.N. Weinstein led the overall team and played roles in experimental and computational aspects of the project as well as in the writing.

## Figures and Tables

**Table 1:** Data fields in AbMiner. Fields that can be searched using AbMiner's Advanced Search function are indicated.

	Fields in AbMiner	Search Field
Antibody Information	Antibody Name	X
	Molecular Weight Range/KDa	X
	Application(s)	X
	Species Reactivity	X
	Ig Isotype	X
	Immunogen Species	X
	Immunogen	
	Epitope	
	Monoclonal/polyclonal	

Gene/Identifiers	Gene Symbol	X
	Gene Name	X
	Symbol status	
	UniGene Cluster Id	X
	Entrez Gene Id	X
	RefSeq (mRNA)	X
	RefSeq (protein)	X

Antibody Validation	Validation Status	
	Band Result	
	Validation Date	
	Dilution Used	

Product Information	Vendor	
	Catalog no	
	Clone designation	
	Lot #	
	Concentration	
	Recommended start dilution	

**Table 2:** Identifiers matched to AbMiner antibodies and the number of antibodies with each identifier.

**AbMiner Gene Information Records**	
Identifier	Number of antibodies

Total antibodies in collection	635
Name (gene symbol)	513
Accession # (Refseq)	778
Unigene Cluster ID	522

**Table 3:** Antibodies in AbMiner with matching UniGene Cluster IDs in four microarray platforms

**Number of antibodies in AbMiner that match microarray identifiers**	
Microarray	Number of antobodies

Antibodies with UniGene IDs	635
Oligo 6.8 K	370
cDNA	430
U95	498
U133	515
